# Associations of individual, household and environmental characteristics with carbon dioxide emissions from motorised passenger travel

**DOI:** 10.1016/j.apenergy.2012.11.001

**Published:** 2013-04

**Authors:** Christian Brand, Anna Goodman, Harry Rutter, Yena Song, David Ogilvie

**Affiliations:** a Environmental Change Institute, School of Geography and the Environment, University of Oxford, United Kingdom; b Faculty of Epidemiology and Population Health, London School of Hygiene and Tropical Medicine, United Kingdom; c National Obesity Observatory, Oxford, United Kingdom; d Faculty of Engineering and the Environment, University of Southampton, United Kingdom; e Medical Research Council Epidemiology Unit, UKCRC Centre for Diet and Activity Research (CEDAR), Institute of Public Health, Cambridge, United Kingdom

**Keywords:** Transport, CO_2_, Climate change, Motorised travel, Socio-economic factors, Environmental factors

## Abstract

Carbon dioxide (CO_2_) emissions from motorised travel are hypothesised to be associated with individual, household, spatial and other environmental factors. Little robust evidence exists on who contributes most (and least) to travel CO_2_ and, in particular, the factors influencing commuting, business, shopping and social travel CO_2_. This paper examines whether and how demographic, socio-economic and other personal and environmental characteristics are associated with land-based passenger transport and associated CO_2_ emissions. Primary data were collected from 3474 adults using a newly developed survey instrument in the iConnect study in the UK. The participants reported their past-week travel activity and vehicle characteristics from which CO_2_ emissions were derived using an adapted travel emissions profiling method. Multivariable linear and logistic regression analyses were used to examine what characteristics predicted higher CO_2_ emissions. CO_2_ emissions from motorised travel were distributed highly unequally, with the top fifth of participants producing more than two fifth of emissions. Car travel dominated overall CO_2_ emissions, making up 90% of the total. The strongest independent predictors of CO_2_ emissions were owning at least one car, being in full-time employment and having a home-work distance of more than 10 km. Income, education and tenure were also strong univariable predictors of CO_2_ emissions, but seemed to be further back on the causal pathway than having a car. Male gender, late-middle age, living in a rural area and having access to a bicycle also showed significant but weaker associations with emissions production. The findings may help inform the development of climate change mitigation policies for the transport sector. Targeting individuals and households with high car ownership, focussing on providing viable alternatives to commuting by car, and supporting planning and other policies that reduce commuting distances may provide an equitable and efficient approach to meeting carbon mitigation targets.

## Highlights

► We examine CO_2_ emissions from motorised passenger travel of 3,474 adults. ► We develop improved methodology for estimating CO_2_ from motorised travel. ► We found highly unequal distributions of CO_2_ emissions. ► Car ownership, employment and home-work distance were strongest predictors of CO_2_. ► Gender, age, urban/rural and bicycle access showed weaker associations.

## 1 Introduction

The transport sector is a major source of unsustainable energy use currently contributing 20–25% of global carbon dioxide (CO_2_) emissions and with its global share projected to rise to 30–50% by 2050 [1]. For transport, CO_2_ is by far the most important greenhouse gas, comprising approximately 99% of direct greenhouse gas emissions [2].^2^ In the UK, total domestic CO_2_ emissions were 590 million tonnes of carbon dioxide (MtCO_2_) in 1990, of which 120 MtCO_2_ (20%) were from the transport sector [2]. By 2009, total CO_2_ emissions were 20% below this level at 474 MtCO_2_, but as domestic transport emissions stayed roughly constant its share rose to 26% by 2009. Of this, cars and taxis accounted for more than half in 2009 (58%) at 70 MtCO_2_, or 15% of all UK domestic CO_2_ emissions.

Reducing CO_2_ emissions by reducing the frequency and volume of car travel is likely to form a key component of a successful strategy to reduce overall CO_2_ emissions [3–6] alongside other approaches such as increased vehicle efficiency and fuel switching. However, efforts to reduce the domination of the car in Western societies have thus far met with limited success [7–9]. Replacing car trips with low carbon modes such as walking, cycling and local public transport is increasingly recognised as important in low carbon strategies [10–14], with further substantial public health benefits [15]. Research from the Sustainable Travel Town demonstration projects in England suggests that about half of all trips currently made by car in urban areas could in principle be shifted to walking, cycling or public transport [16,17]. Knowledge of which individuals are responsible for disproportionately high levels of emissions can promote effective carbon reduction while reducing the socially divisive and inequitable effects of a transport system dominated by less sustainable modes [18–20].

There has been much research on the determinants of travel behaviour in general, and trip distances in particular, suggesting that travel patterns vary according to demographic, socio-economic, cultural and lifestyle characteristics [21–26]. However, there is still little evidence of the distribution and composition of CO_2_ emissions arising from such travel activity at the disaggregate (i.e. individual, household, local) level. There is some evidence that while mode choice, income, employment status, housing tenure and car ownership are significantly and strongly associated with overall emissions, factors related to accessibility, household location and gender are not once controlled for key demographic and socio-economic factors [27–30].

This paper aims to narrow this gap in the literature by describing the development of improved methods for estimating CO_2_ emissions from motorised travel that (a) allow investigation of emissions by journey purpose, transport mode and vehicle technology and (b) are independent of whether the individuals concerned are drivers or passengers. Using primary cross-sectional data collected in a large population survey across three case study sites in the UK, this paper also aims to explore how demographic and socio-economic position and other personal characteristics are associated with carbon emissions from motorised travel.

## 2 Methods

### 2.1 Study population

Our analyses use baseline cross-sectional data from the *iConnect* study (www.iconnect.ac.uk), which seeks to examine the effects of new transport infrastructure on travel, physical activity and CO_2_ emissions [31,32]. A total of 22,500 adults in three areas of the UK (the study sites at Cardiff, Kenilworth and Southampton) were randomly selected from the edited electoral register in April 2010. Individuals were posted a survey pack containing an information sheet, questionnaire and consent form, and were asked to return the consent form and questionnaire in the pre-paid envelope provided. Participants who did not return questionnaires within two weeks were sent a second survey pack. The University of Southampton Research Ethics Committee granted ethical approval (CEE200809-15).

In total, 3,516 individuals returned survey packs (a 16% response rate). Participants who did not report any travel in the past week (*N* = 42) were excluded from the analyses. The resulting study population comprised 3,474 individuals (age range 18–96, 55% female).

### 2.2 CO_2_ emissions calculations

A detailed seven-day recall survey instrument was used to assess travel activity for five journey purposes: to and from work; to and from school or place of study; in the course of business; for shopping and personal business; and for visiting friends or relatives or other social activities. The entire questionnaire has been reproduced in Ogilvie et al. [32]. For each journey purpose, respondents were asked to recall the total number of journeys made and the total time spent and distance travelled by seven modes: walking, cycling, bus, train, car (as driver), car (as passenger) and ‘other’. If only distance or time was reported then the counterpart was imputed using the mean observed speed for each mode and journey purpose. For bus, train and car travel, mean speeds were also used to impute time if the average speed was otherwise implausibly slow (<3 miles/h across more than 2 h, or <10 miles/h across more than 10 h) or implausibly fast (>120 miles/h).

**Fig. 1:**
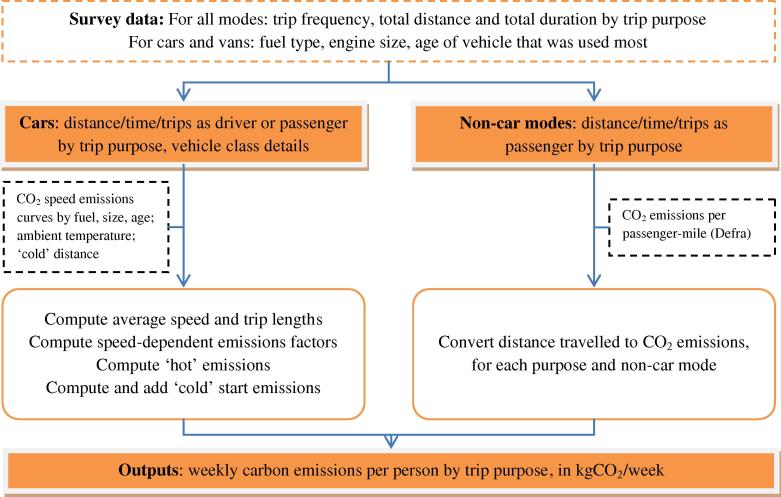
CO_2_ emissions calculation methods for cars and other motorised modes.

As described fully in Appendix A, we used these travel activity data to derive CO_2_ emissions.^3^ The methods differed for car and non-car modes. First, for travel by bus, train and ‘other’ modes (mainly taxi, motorcycle and van), self-reported data on distance travelled by trip purpose were multiplied by mode-specific, average CO_2_ emissions factors obtained from DEFRA [33] (Fig. 1, right-hand side).^4^ Second, for household cars and vans, the self-reported data on trip frequencies and duration as well as vehicle fuel, size and age allowed for the use of a more disaggregate method (Fig. 1, left-hand side). This included the estimation of ‘hot’ CO_2_ emissions (when the engine is running at operating temperature) using speed-emissions curves developed for the Department for Transport [34] and ‘cold’ CO_2_ emissions (excess emissions due to suboptimal fuel combustion during the warm-up phase). Emissions from travel ‘to and from work’ and ‘to and from school or place of study’ were combined into a ‘commuting’ category. As we lacked detailed data on car-sharing we modelled CO_2_ in two ways, (a) one dividing emissions from car travel between passengers and drivers and (b) one assigning all emissions to the driver. The substantive findings were generally identical and we therefore report in the main text the results for CO_2_ divided between drivers and passengers (see Appendix A for further details and Appendix B for results replicated for the driver-only approach).

### 2.3 Individual, household and environmental predictor variables

Table 1 shows the individual, household and environmental variables we examined as predictors of transport carbon emissions. Of these, the following were self-reported: sex, age, ethnicity, presence of any child aged under 16 in the household, highest educational qualification, annual household income, housing tenure, employment status and availability of any adult bicycle in the household. Cars per adult in the household were calculated based on self-reported numbers of cars and adults (aged 16 or over) in the household. By matching home postcodes to Lower Super Output Areas (LSOAs) we assigned urban/rural status and population density, using mid-2010 population estimates for the latter [35]. Home-work distance was calculated as the shortest road network distance from the home postcode to the work postcode; those reporting no fixed workplace (*N* = 43) were combined with those reporting a commute distance of over 20 km because this was the group with the most similar total weekly commute distance. Home-retail distance was calculated as the shortest network distance to the nearest Retail Core in 2004 [36].

### 2.4 Statistical analysis

We used linear regression to examine the predictors of transport-related carbon emissions for all journey purposes and for different types of journey, weighting participants by the age and sex profile of their LSOA in 2009 [37]. Because CO_2_ emissions were positively skewed, we applied the transformation ‘log([*x*/mean(*x*)] + 0.01)’ (adding 0.01 to avoid turning zeros into missing values) and then standardised these log-transformed outcomes. We fitted single-level regression models because fitting multi-level models indicated that spatial clustering was low (e.g. 2.6% variation in log-transformed CO_2_ explained at site level) and did not affect our substantive findings. As a sensitivity analysis, we also present in Appendix C the results of logistic regression analyses predicting the binary variable of being in the ‘top 20%’ of carbon emitters.

As the percentage of missing data for our explanatory variables ranged from 0% to 17%, we used multiple imputation by chained equations (5 imputations) to impute missing values under an assumption of missing at random, including in the imputation model all covariates and outcomes ever entered in the regression models. Our main substantive findings were unchanged in sensitivity analyses which used complete case analyses or which excluded the two predictors with more than 6% missing data (adult bicycle access and income). We used a hierarchical approach to building multivariable regression models [38], starting with socio-demographic variables which we hypothesised to be further back on the causal pathway and then proceeding to add environmental variables and finally variables relating to car/bike access. Age and commute distance showed evidence of non-linearity in univariable analyses (both *p* < 0.001 for linearity, as judged by including a quadratic term), and we therefore entered these as categorical variables and present *p*-values for heterogeneity. By contrast population density and home-retail distance showed no evidence of non-linearity (*p* > 0.1 in both univariable and multivariable analyses) and so were entered as continuous terms. All analyses used Stata 11 except the calculation of home-work and home-retail distances which used ArcGIS 9.

## 3 Results and discussion

### 3.1 Levels and sources of carbon emissions from motorised travel

As shown in Table 1 above, 55% of our sample was female, 95% white and slightly older than the local populations (51% female, 91% white, 18% were 65 years or older based on District level population estimates for mid 2010) [37]. Car ownership in our sample was likely to be higher than for local populations, with only 15% of respondents stating they did not have access to a car compared to 23% and 21% of households not owning a car or van in England (excluding London) and Wales [2] respectively.

Within our sample mean carbon emissions from all motorised surface passenger travel were 35.1 kg of CO_2_ (kgCO_2_) per person per week. This corresponds to about 1.6 tonnes of CO_2_ (tCO_2_) per person per year^5^, a figure comparable to government estimates of per capita road transport emissions of 2.2 tCO_2_, once emissions from road freight (about 30% of road transport emissions in Great Britain) are discounted [39,40]. The above mean was substantially higher than the derived median (18.8 kgCO_2_ per person per week) and near the upper end of the derived interquartile range (6.2–42.0 kgCO_2_ per person per week), suggesting a highly skewed distribution of emissions. In other words, a small proportion of individuals were responsible for most of the emissions, with the bottom fifth producing 0.8% of emissions and the top fifth 63%. Interestingly, the distribution was quite similar when allocating all car travel emissions to the driver in our sensitivity analysis, with the bottom fifth producing 0.2% of emission and the top fifth 65%.

**Fig. 2:**
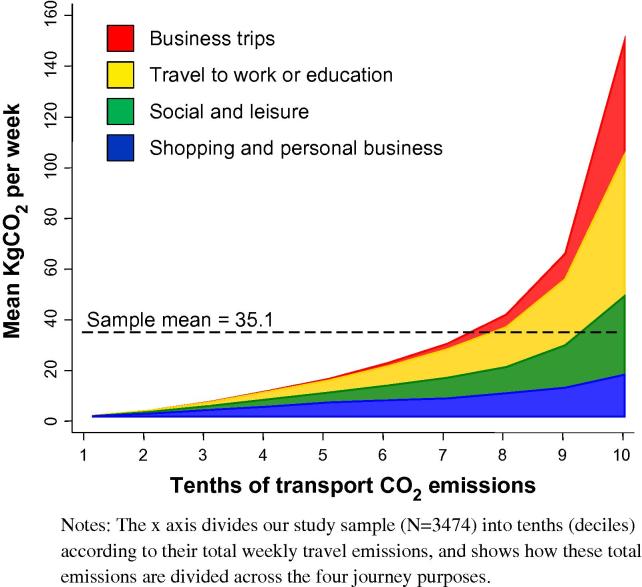
Distributions of CO_2_ emissions by travel emissions decile, subdivided by journey type. Notes: The *x* axis divides our study sample (*N* = 3474) into tenths (deciles) according to their total weekly travel emissions, and shows how these total emissions are divided across the four journey purposes.

While travel to and from work produced the largest share of CO_2_ emissions (35%), there were also considerable contributions from social trips (24%), business trips (19%) and travel for shopping or personal business (19%). Travel to and from school or place of study showed a relatively low share of 3% of total emissions. This reflected the lower reported frequencies for these education trip purposes (only 12%, *N* = 414, reported making at least one trip for education in the past week, vs. 20% for business, 52% for work, 65% for social and 77% for shopping) and the shorter average travel distances involved (mean 33 km/week for those making any such trip, versus 36 km for shopping, 65 km for social, 101 km for work and 145 km for business). It may also in part reflect allocation of the ‘main purpose of a trip’ to other purposes in trip chains. We therefore combined work and education trips into a single category of ‘commuting trips’. Again, the distributions were skewed towards a small minority producing a large share of the total. Emissions from shopping and personal business trips were the most equally distributed; those from business and commuting trips the least equally (Fig. 2).

Car travel dominated overall emissions from motorised travel (90% of total), followed by train (4%), bus (4%), other private transport (e.g. taxi, van, motorcycle: 1.6%), and other public transport (e.g. underground, coach, ferry: 0.3%). Among our three case study sites, respondents in Southampton produced markedly lower average CO_2_ emissions (median 12.1 kgCO_2_ per week, of which cars generated 86%), while those in Kenilworth produced higher emissions that were even more dominated by those from car use (median 23.8 kgCO_2_ per week, of which cars generated 91%). This geographical discrepancy is in line with regional per capita CO_2_ emissions estimates [39:2008data] and can partially be explained by the different demographics: for example the Southampton sample was younger and included more students than the Kenilworth sample.

Although markedly unequal, the levels and distributional characteristics of total CO_2_ emissions are in line with previous studies using similar methods [27–29]. The intriguing question of what characteristics predict higher emissions is explored next.

### 3.2 Associations and predictors of carbon emissions from motorised travel

The individual and environmental predictors of CO_2_ emissions from motorised travel are shown in Table 2 (for total CO_2_) and Table 3 (for CO_2_ by trip purpose). The minimally-adjusted analyses suggested that most of the individual, environmental and car access variables were significantly related to CO_2_ emissions production. The strongest and most significant associations emerged between CO_2_ emissions and income, tenure, employment status, education, home-work distance and car availability. Some of the other environmental (in particular site, urban/rural) and demographic variables (gender, age, any child under 16) were moderately and significantly related to total CO_2_ production. After adjusting for individual and environmental characteristics in the multivariable models both significance and strength of the associations between predictors and emissions changed somewhat, as discussed below across the four domains of analysis.

#### 3.2.1 Demographic characteristics

There was evidence that male gender was associated with higher total CO_2_, with median emissions of 23.7 kgCO_2_ per week among men vs. 15.7 kgCO_2_ among women, and with total CO_2_ emissions among men being 0.15 standard deviations (SD) higher than those among women (95% CI 0.06, 0.23: Table 2, multivariable model 1). This effect was somewhat attenuated after adjusting for environmental variables and car and bike access (model 3), but there remained evidence of an independent effect (*p* < 0.01 for heterogeneity). Men were also more likely to fall into the ‘top 20% emitters’ category (28% of men vs. 14% of women: Table C.1, Appendix C). This gender gap seemed partly to reflect the fact that men in our sample were more likely than women to be in full-time paid work (48% vs. 35%). Further multivariable analysis of CO_2_ emissions for different trip purposes (Table 3) showed, however, that men and women did not differ in emissions relating to commuting, shopping/personal business or social/leisure trips. Instead higher emissions in men were entirely (and literally) driven by higher travel activity in men on business trips – a finding in line with results from a previous study [41]. Interestingly, car *availability* (unlike usage) was equally distributed amongst men and women: 86% of men and 85% of women had access to a car in their household.

Furthermore, there was some evidence of higher CO_2_ emissions for those in the middle age range (35–64 years), with median emissions about twice as high as those of younger (18–34) or older (65+) participants. While this effect was again substantially attenuated after adjusting for socio-demographic (model 1), environmental (model 2) and car/bike access (model 3) variables, there remained evidence of an independent effect (*p* < 0.01 for heterogeneity). For example, in the fully adjusted analysis log-transformed emissions among respondents aged 50–64 years were 0.20 SD higher than for younger (18–34) people (95% CI 0.09, 0.31). There was no evidence that non-white ethnicity predicted average CO_2_ emissions totals (Table 2) but some evidence that non-white individuals were overrepresented among the top 20% of emitters (25% of non-whites vs. 20% of whites: Appendix C, Table C.1). Also, while non-white individuals showed significantly higher emissions from commuting, they were responsible for significantly lower emissions from social and leisure trips (Table 3). By contrast, after adjusting for other socio-demographic characteristics, there was no evidence of an independent effect of having children under 16.

#### 3.2.2 Socio-economic characteristics

**Fig. 3:**
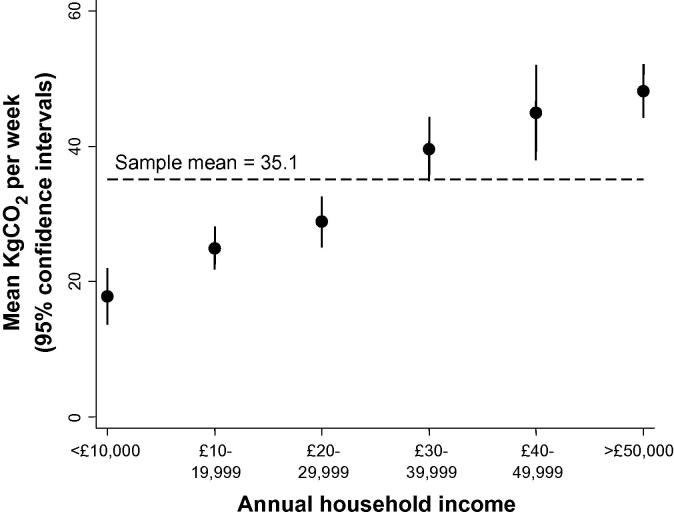
Mean CO_2_ emissions from all travel by household income band, car CO_2_ allocated between driver and passengers (*N* = 3474).

The minimally-adjusted analysis suggested that household income was strongly associated with total CO_2_ emissions, with log-transformed emissions among individuals on higher incomes (>£40,000 per year) being 0.71 standard deviations (SDs) higher than for those on lower incomes (<£20,000) (95% CI 0.83, 0.59; Table 2). This is further illustrated in Fig. 3 showing mean CO_2_ emissions rising steadily with higher incomes – a result which echoes other studies linking travel patterns and environmental effects [21], travel activity, fuel use and income [42], and energy consumption and income [43].

The strong and positive effects of *socio-economic* characteristics (education, income, housing tenure, employment status) diminished somewhat but remained strong after adjusting for all socio-economic position (SEP) indicators (model 1), with evidence of higher CO_2_ emissions for individuals with a degree, on higher incomes, owning a house and in full-time employment. For example, for housing tenure, CO_2_ emissions were 0.53 SD higher (95% CI 0.37, 0.69) for respondents owning their house than for those living in council rented accommodation. Furthermore, employment status was strongly associated with total CO_2_ in the minimally-adjusted analysis, with respondents in full-time employment producing emissions which were 0.26 SD higher than part-time workers, around 0.5 SD higher than retired individuals or those looking after home and family, and 0.90 SD higher than students. This association remained strong and significant in the adjusted models, with further evidence that workers were overrepresented among the top 20% of emitters (33% of full-time workers vs. 17% of part-time workers vs. 7% students vs. 11% retired: Appendix C, Table C.1).

These socio-economic associations changed little after additionally adjusting for the environmental variables (model 2) except that adjusting for commute distance attenuated the regression coefficients associated with not working. The socio-economic effects were, however, markedly attenuated towards the null (and often completely to the null, except for employment status) after also adjusting for car access (model 3). This suggested that income and working status were predictors but appeared to be further back on the causal pathway than having a car. In other words, it appeared that the effect of high SEP might in part be due to income or employment status affecting people’s ability or need to buy a car, and this in turn affected their CO_2_ emissions.^6^ Interestingly, when disaggregating emissions by journey purpose, there was evidence in the full multivariable model that workers were responsible for *higher* CO_2_ emissions for commuting and business, but *lower* emissions for shopping/personal business and social/leisure journeys when compared to non-workers – except students who showed significantly lower emissions across all the journey purposes.

#### 3.2.3 Spatial and environmental characteristics

In the minimally-adjusted analysis, home-work distance proved to be strongly associated with CO_2_ production, with CO_2_ emissions being 0.92 SD (95% CI 0.76, 1.08) higher for respondents living more than 20 km from their place of work or study than for those living only 2–5 km away. Even after adjusting for demographic, socio-economic and other environmental variables, home-work distance proved to be a strong predictor of CO_2_ emissions. For example, CO_2_ emissions were 0.77 SD (95% CI 0.63, 0.91) higher for respondents living more than 20 km from their place of work than for those living only 2–5 km away. A home-work distance of more than 10 km also considerably increased the likelihood of falling into the top 20% of emitters (62% for >20 km vs. 35% for 10–20 km vs. 11–18% for <10 km: Appendix C, Table C.1). As expected, these associations with commuting distance were confined to commuting emissions, with no association found with other trip purposes (Table 3). On the other hand, home-retail centre distance was not significant even for shopping/personal business in the full model.

There was marginal evidence that respondents living in Kenilworth had higher emissions than those living in Cardiff or Southampton, even after adjusting for demographic, socio-economic and other environmental characteristics (model 2). This may reflect poorer accessibility to public transport, the relative price inelasticity of rural households [19] and the fact that there is no rail station in Kenilworth with access to nearby major employment centres. However, the difference in emissions by site was relatively small in these fully-adjusted analyses, suggesting that the model already included the variables that explain most of the inter-site difference in mean levels.

#### 3.2.4 Availability of cars, vans and bicycles

Respondents who had one or more cars per adult in the household showed considerably higher average emissions than people with less than one car per adult (0.52 SD), and substantially higher emissions overall than those with no cars in the household (0.52 + 0.97 = 1.49 SD).

Car availability remained a very strong predictor in all multivariable models, and adjusting for this in model 3 reduced – in some cases to the null – the effects of higher SEP such as education, income and tenure. However, the effects of both employment status and home-work distance remained strong and highly significant, even after adjusting for car access (model 3). There was also strong evidence that people with access to a car were substantially overrepresented among the top 20% of emitters (29% vs. 16% vs. 4%: Appendix C, Table C.1). Interestingly, CO_2_ emissions for respondents without car access were much lower than for people with at least one car available to them for all trip purposes except for business journeys, for which the association was much weaker. Moreover, while bicycle access was not associated with carbon emissions in the minimally-adjusted analyses, it became moderately associated with lower emissions levels after adjusting for the fact that bicycle access was much higher among those of high SEP (e.g. 76% of those with a household income of >£40,000 per year had at least one adult bicycle available for use, versus 42% of those with an income of <£20,000).

Finally, it is worth noting that the full model (model 3) was much better at predicting emissions from commuting (*R*^2^ = 0.54) than from business, shopping or social travel (*R*^2^ ⩽ 0.18). This is perhaps not that surprising given the covariates included in the model.

### 3.3 Sensitivity analysis: comparison of the two emissions allocation methods for cars

We compared Table 2 above, which used the ‘driver/passenger’ method of allocating emissions for cars, with Table B.1 in Appendix B, which used the ‘driver only’ method. The two methods for assigning emissions from cars generally gave very similar or identical findings *except* that the effect of gender was approximately doubled in the ‘driver only’ method. Specifically, when looking at the analyses by journey purpose, a strong association between CO_2_ emissions and male gender persisted for business travel but also appeared for ‘shopping and personal business’ and ‘social and leisure’ travel. This suggests that much of the gender difference in the ‘driver only’ method may not reflect a real difference in the extent to which individuals make decisions to travel by car. Instead it may be an *artefact* of personal or household preferences for driving a car in multi-occupancy trips. The ‘driver/passenger’ method, in our view, produces more accurate (and conservative) estimates of social and demographic differences in CO_2_ emissions as it allocates emissions equally to both drivers and passengers. It also allowed us to investigate more directly whether the tendency for men to drive rather than be the passenger was the sole explanation for the apparent gender gap in CO_2_ emissions or whether this gender gap also reflected other differences in travel behaviour. It was precisely this thinking that led us to favour the ‘driver/passenger’ method for the main analysis as a novel methodological alternative for studies working with imperfect data collection methods.

### 3.4 Limitations to the study

In interpreting these findings it is important to bear in mind this study’s limitations. First, the 16% response rate means that our sample cannot be assumed to be representative. In particular our sample may be more car dependent than the general population: in our sample, only 15% of participants said they did not have access to a car, versus 25% of households nationally [44]. However, past-week travel behaviour in our sample was similar to that reported nationally: for example, in our sample 79% of travel distance was covered by car, 16% by other modes and 5% by active travel, versus 78%, 18% and 4% respectively in the 2010 National Travel Survey [44]. This is likely to be the reason why the resultant travel emissions are comparable to national averages [2,39]. Moreover, even if our sample is biased with respect to car availability, we know of no reason to expect this to bias the *associations* between these variables. A second key limitation is that our data are cross-sectional, meaning that the direction of causality (if any) behind many of the observed associations is unclear. Third, the interdisciplinary breadth of the iConnect study meant that we measured travel behaviour, vehicle and spatial-environmental characteristics using briefer survey tools than might have been feasible in a single-discipline study. This may have introduced some measurement error that could have attenuated our effect sizes. Fourth, the observation that our regression models did not account for more than 54% of the variation in the population suggests that CO_2_ emissions are also influenced by other factors such as lifestyle and socio-cultural factors, as shown by a number of studies [25,45]. Finally, we recognise that we cannot make robust policy recommendations based on strength of associations alone. Other key considerations are the frequency of a characteristic (e.g. a rare characteristic with a strong effect may have less of an impact at the population level than a more common characteristic with a medium effect) and amenability of a characteristic to modification and intervention (e.g. home-work distance may not be as easily targeted as, say, car ownership or usage through pricing mechanisms).

## 4 Conclusions

This paper started by noting that there is little evidence from robust studies exploring the disaggregate distributions of CO_2_ emissions from land-based passenger travel and identifying the demographic, socio-economic and environmental predictors of those emissions. It aimed to narrow this gap in the literature by developing improved and robust methods for estimating CO_2_ emissions and applying these methods in a cross-sectional study collecting detailed data on travel activity by mode and journey purpose, vehicle ownership and vehicle use. The innovations of this study lie in an improved method, a new cross-sectional dataset and new evidence of associations of individual, household and environmental characteristics with carbon emissions from motorised passenger travel.

The analysis of a sample of nearly 3500 adults across the three sites confirms the highly unequal distributions of carbon emissions from motorised travel found elsewhere [28,29,46,47], with the top 20% producing 63% of total emissions. The findings that CO_2_ emissions were strongly associated with car availability, employment status and home-work distance—and less so with other significant factors such as gender, age, income, urban/rural and bicycle access—may help inform the development of efficient and equitable transport and climate change policy. Although more detailed further longitudinal analysis is warranted to clarify the direction of causality underlying some of these associations, this work suggests that targeting the high emitters may provide an equitable and efficient approach to meeting carbon mitigation targets.

## Figures and Tables

**Table B.1:** Individual and environmental predictors of total transport CO_2_ emissions, car CO_2_ allocated to driver only (*N* = 3474).

Variable	Level	Median	Regression coefficients (βs) and 95% CI for standardised log-transformed carbon			
Min-adjusted^a^	Multivariable 1	Multivariable 2	Multivariable 3
	*R*^2^ = 0.22	*R*^2^ = 0.29	*R*^2^ = 0.37
Sex	Female	14.3	0⁎⁎⁎	0⁎⁎⁎	0⁎⁎⁎	0⁎⁎⁎
Male	24.2	0.25 (0.17, 0.34)	0.22 (0.14, 0.31)	0.18 (0.10, 0.26)	0.21 (0.14, 0.28)

Age	18–34 years	12.9	0⁎⁎⁎	0⁎⁎⁎	0⁎⁎⁎	0⁎⁎⁎
35–49 years	26.8	0.42 (0.30, 0.53)	0.16 (0.04, 0.28)	0.14 (0.03, 0.25)	0.13 (0.03, 0.24)
50–64 years	22.5	0.32 (0.21, 0.43)	0.22 (0.09, 0.34)	0.25 (0.12, 0.37)	0.23 (0.11, 0.35)
>65 years	11.9	−0.18 (−0.30, −0.06)	0.02 (−0.15, 0.19)	0.05 (−0.12, 0.22)	0.06 (−0.11, 0.22)

Ethnicity	White	18.0	0	0	0	0
Non-White	16.5	−0.10 (−0.32, 0.12)	0.06 (−0.16, 0.28)	0.10 (−0.11, 0.31)	0.14 (−0.05, 0.33)

Any child under 16	No	16.2	0⁎	0	0	0
Yes	24.6	0.15 (0.04, 0.27)	0.08 (−0.03, 0.19)	0.09 (−0.01, 0.20)	0.04 (−0.06, 0.14)

Education	Degree	24.6	0⁎⁎⁎	0⁎⁎⁎	0⁎⁎⁎	0⁎⁎⁎
A-level	17.8	−0.34 (−0.47, −0.22)	−0.15 (−0.28, −0.03)	−0.14 (−0.26, −0.02)	−0.12 (−0.23, −0.01)
GCSE	16.7	−0.37 (−0.50, −0.25)	−0.23 (−0.35, −0.11)	−0.23 (−0.34, −0.12)	−0.14 (−0.24, −0.04)
No formal	10.5	−0.58 (−0.69, −0.47)	−0.33 (−0.45, −0.22)	−0.31 (−0.42, −0.20)	−0.23 (−0.34, −0.12)

Annual household income	>£40,000	31.4	0⁎⁎⁎	0⁎⁎	0⁎	0
£20–40,000	21.7	−0.22 (−0.33, −0.11)	−0.10 (−0.21, 0.02)	−0.03 (−0.14, 0.07)	−0.02 (−0.12, 0.09)
<£20,000	9.5	−0.64 (−0.75, −0.53)	−0.26 (−0.38, −0.14)	−0.18 (−0.29, −0.06)	−0.11 (−0.22, 0.00)

Housing tenure	Owned	21.9	0⁎⁎⁎	0⁎⁎⁎	0⁎⁎⁎	0
Privately rented	7.8	−0.51 (−0.65, −0.36)	−0.33 (−0.47, −0.18)	−0.17 (−0.32, −0.03)	−0.07 (−0.20, 0.06)
Council rented	3.4	−0.82 (−0.97, −0.68)	−0.44 (−0.60, −0.29)	−0.35 (−0.51, −0.20)	−0.07 (−0.24, 0.09)
Other	14.3	−0.10 (−0.35, 0.16)	0.05 (−0.17, 0.26)	0.05 (−0.16, 0.27)	0.13 (−0.06, 0.33)

Employment status	Full-time	30.5	0⁎⁎⁎	0⁎⁎⁎	0⁎⁎⁎	0⁎⁎⁎
Part-time	21.5	−0.18 (−0.28, −0.08)	−0.14 (−0.24, −0.04)	−0.07 (−0.17, 0.03)	−0.07 (−0.16, 0.02)
Student	2.6	−0.83 (−1.03, −0.63)	−0.67 (−0.88, −0.46)	−0.62 (−0.82, −0.42)	−0.45 (−0.64, −0.26)
Retired	12.5	−0.47 (−0.61, −0.33)	−0.37 (−0.50, −0.24)	−0.13 (−0.29, 0.03)	−0.14 (−0.30, 0.02)
Home duties	12.3	−0.53 (−0.73, −0.32)	−0.43 (−0.62, −0.23)	−0.27 (−0.48, −0.06)	−0.20 (−0.39, 0.00)
Other	3.4	−1.00 (−1.18, −0.82)	−0.67 (−0.88, −0.47)	−0.46 (−0.67, −0.25)	−0.36 (−0.56, −0.15)

Site	Southampton	10.7	0⁎⁎⁎		0	0
Cardiff	18.9	0.22 (0.12, 0.32)		0.01 (−0.11, 0.13)	0.02 (−0.10, 0.13)
Kenilworth	23.5	0.40 (0.29, 0.50)		0.10 (−0.02, 0.23)	0.11 (−0.01, 0.23)

Urban/rural status	Urban	17.1	0⁎⁎⁎		0	0
Rural	33.3	0.42 (0.27, 0.58)		0.18 (0.00, 0.37)	0.14 (−0.03, 0.32)

Population density	Change per 10 people per hectare		−0.05 (−0.06, −0.03)⁎⁎⁎		−0.02 (−0.03, 0.00)⁎	−0.01 (−0.02, 0.00)

Home-work distance	0–2 km/no comm.	9.6	−0.18 (−0.31, −0.05)		−0.04 (−0.18, 0.09)	−0.05 (−0.18, 0.08)
2–5 km	13.1	0⁎⁎⁎		0⁎⁎⁎	0⁎⁎⁎
5–10 km	25.7	0.39 (0.22, 0.55)		0.26 (0.09, 0.42)	0.20 (0.05, 0.35)
10–20 km	36.7	0.74 (0.59, 0.89)		0.56 (0.42, 0.69)	0.45 (0.33, 0.58)
⩾20 km or variable	65.2	0.83 (0.68, 0.98)		0.71 (0.57, 0.85)	0.61 (0.47, 0.75)

Home-retail distance	Change per kilometer		0.07 (0.05, 0.09)⁎⁎⁎		−0.01 (−0.03, 0.02)	−0.02 (−0.04, 0.01)

Cars per adult in household	No cars	1.5	−0.79 (−0.91, −0.67)			−0.60 (−0.73, −0.47)
<1 Car per adult	13.5	0⁎⁎⁎			0⁎⁎⁎
⩾1 Cars per adult	28.5	0.57 (0.49, 0.65)			0.39 (0.32, 0.47)

Any adult bike	No	14.9	0			0⁎⁎
Yes	20.8	0.01 (−0.08, 0.10)			−0.11 (−0.19, −0.03)

⁎ *p* < 0.05.

⁎⁎ *p* < 0.01.

⁎⁎⁎ *p* < 0.001.

a Minimally-adjusted analyses adjust for age and sex only, multivariable analyses adjust for all variables in column. Outcome is standardised log-transformed CO_2_ (no unit due to transformation to log([CO_2_/mean(CO_2_)] + 0.01)).

**Table C.1:** Individual and environmental predictors of ‘high’ transport carbon emissions, car CO_2_ allocated between drivers and passengers (*N* = 3474).

Variable	Level	Percent	Odds ratio (95% CI) for being in top 20% of transport carbon emissions			
Min-adjusted^a^	Multivariable 1	Multivariable 2	Multivariable 3
	*R*^2^ = 0.13	*R*^2^ = 0.21	*R*^2^ = 0.24
Sex	Female	14	1^∗∗∗^	1^∗∗∗^	1^∗∗∗^	1^∗∗∗^
Male	28	2.55 (2.08, 3.13)	2.32 (1.86, 2.90)	2.07 (1.64, 2.61)	2.28 (1.79, 2.89)

Age	18–34 years	18	1^∗∗∗^	1^∗^	1	1
35–49 years	28	1.73 (1.32, 2.28)	1.57 (1.14, 2.17)	1.61 (1.14, 2.28)	1.61 (1.14, 2.27)
50–64 years	22	1.16 (0.88, 1.51)	1.26 (0.91, 1.74)	1.46 (1.02, 2.09)	1.44 (1.01, 2.06)
>65 years	13	0.51 (0.38, 0.70)	1.37 (0.86, 2.19)	1.60 (0.93, 2.73)	1.55 (0.90, 2.68)

Ethnicity	White	20	1	1^∗^	1^∗^	1^∗∗^
Non-White	25	1.22 (0.79, 1.90)	1.75 (1.07, 2.86)	1.92 (1.14, 3.23)	2.14 (1.26, 3.63)

Any child under 16	No	19	1	1	1	1
Yes	24	0.89 (0.68, 1.17)	0.75 (0.55, 1.02)	0.78 (0.54, 1.11)	0.72 (0.51, 1.03)

Education	Degree	26	1^∗∗∗^	1^∗^	1^∗^	1
A-level	22	0.76 (0.57, 1.02)	1.03 (0.76, 1.41)	1.02 (0.73, 1.43)	1.03 (0.74, 1.43)
GCSE	19	0.60 (0.45, 0.80)	0.84 (0.62, 1.15)	0.78 (0.56, 1.08)	0.87 (0.62, 1.22)
No formal	12	0.40 (0.30, 0.53)	0.62 (0.46, 0.85)	0.60 (0.43, 0.85)	0.65 (0.46, 0.92)

Annual household income	>£40,000	34	1^∗∗∗^	1^∗∗∗^	1^∗∗^	1∗∗
£20–40,000	21	0.57 (0.44, 0.74)	0.65 (0.50, 0.85)	0.73 (0.56, 0.97)	0.77 (0.58, 1.02)
<£20,000	9	0.26 (0.18, 0.36)	0.42 (0.29, 0.60)	0.49 (0.33, 0.71)	0.53 (0.36, 0.78)

Housing tenure	Owned	23	1^∗∗∗^	1^∗^	1	1
Privately rented	15	0.56 (0.39, 0.80)	0.70 (0.48, 1.03)	1.03 (0.70, 1.52)	1.20 (0.81, 1.77)
Council rented	5	0.16 (0.08, 0.32)	0.35 (0.17, 0.73)	0.39 (0.18, 0.82)	0.52 (0.22, 1.22)
Other	18	0.76 (0.37, 1.56)	0.97 (0.47, 2.03)	0.99 (0.46, 2.14)	1.07 (0.52, 2.19)

Employment status	Full-time	33	1^∗∗∗^	1^∗∗∗^	1^∗∗∗^	1^∗∗∗^
Part-time	17	0.45 (0.34, 0.61)	0.52 (0.39, 0.71)	0.57 (0.41, 0.80)	0.58 (0.42, 0.82)
Student	7	0.39 (0.21, 0.71)	0.43 (0.23, 0.82)	0.35 (0.17, 0.71)	0.48 (0.24, 0.99)
Retired	11	0.26 (0.18, 0.38)	0.32 (0.22, 0.47)	0.44 (0.27, 0.70)	0.44 (0.27, 0.71)
Home duties	12	0.35 (0.20, 0.61)	0.47 (0.27, 0.83)	0.53 (0.27, 1.03)	0.62 (0.32, 1.18)
Other	7	0.13 (0.06, 0.28)	0.22 (0.10, 0.51)	0.28 (0.11, 0.69)	0.31 (0.12, 0.80)

Site	Southampton	15	1^∗∗∗^		1	1
Cardiff	21	1.28 (0.99, 1.64)		1.15 (0.76, 1.73)	1.11 (0.74, 1.68)
Kenilworth	24	1.97 (1.52, 2.56)		1.54 (0.99, 2.38)	1.51 (0.96, 2.37)

Urban/rural status	Urban	20	1^∗∗∗^		1	1
Rural	33	2.24 (1.46, 3.42)		1.60 (0.90, 2.86)	1.57 (0.89, 2.77)

Population density	Change per 10 people per hectare	–	0.94 (0.91,0.98)^∗∗∗^		0.99 (0.95, 1.04)	1.00 (0.95, 1.05)

Home-work distance	0–2 km/no comm.	13	1.06 (0.72, 1.58)		1.48 (0.95, 2.30)	1.48 (0.94, 2.34)
2–5 km	11	1^∗∗∗^		1^∗∗∗^	1^∗∗∗^
5–10 km	18	1.56 (1.01, 2.41)		1.26 (0.79, 2.03)	1.19 (0.74, 1.89)
10–20 km	35	4.29 (2.77, 6.62)		3.39 (2.14, 5.37)	3.01 (1.88, 4.80)
⩾20 km or variable	62	9.08 (6.14, 13.42)		8.32 (5.54, 12.49)	7.58 (5.00, 11.49)

Home-retail distance	Change per kilometers	–	1.10 (1.05,1.16)^∗∗∗^		0.97 (0.90, 1.05)	0.95 (0.88, 1.03)

Cars per adult in household	No cars	4	0.20 (0.11, 0.37)			0.32 (0.17, 0.60)
<1 Car per adult	16	1^∗∗∗^			1^∗∗∗^
⩾1 Cars per adult	29	2.66 (2.11, 3.37)			2.16 (1.67, 2.80)

Any adult bike	No	17	1			1
Yes	23	1.05 (0.84, 1.32)			0.89 (0.68, 1.15)

**Table 1:** Socio-demographic and environmental characteristics of participants.

Domain	Variable	Level	*N* (%)
Demographic	Sex	Female	1903 (55.0)
	Male	1558 (45.0)
Age	18–34 years	792 (23.1)
	35–49 years	802 (23.4)
	50–64 years	991 (28.9)
	>65 years	839 (24.5)
Ethnicity^a^	White	3244 (94.8)
	Asian	105 (3.1)
	Black	26 (0.8)
	Other	47 (1.4)
Any child under 16	No	2722 (79.5)
	Yes	702 (20.5)

Socio-economic	Education	Degree	1374 (40.9)
	A-level	599 (17.8)
	GCSE	630 (18.7)
	No formal	758 (22.6)
Annual household income	>£40,000	1057 (36.8)
	£20,001–40,000	936 (32.6)
	⩽£20,000	878 (30.6)
Housing tenure	Owned	2573 (75.5)
	Privately rented	506 (14.9)
	Council rented	254 (7.5)
	Other	74 (2.2)
Employment status	Full-time	1403 (41.3)
	Part-time	476 (14.0)
	Student	222 (6.5)
	Retired	939 (27.6)
	Home duties	145 (4.3)
	Other	214 (6.3)

Environment	Site	Southampton	1112 (32.0)
	Cardiff	1114 (32.1)
	Kenilworth	1248 (35.9)
Urban/rural status	Urban	3316 (95.5)
	Rural	158 (4.6)
Population density (people per hectare)	<25	1237 (35.6)
	25–50	1231 (35.4)
	⩾50	1006 (29.0)
Home-work distance	0–2 km/No commute	1464 (47.8)
	2–5 km	453 (14.8)
	5–10 km	506 (16.5)
	10–20 km	278 (9.1)
	⩾20 km Or variable	359 (11.7)
Home-retail distance	0–2 km	272 (7.8)
	2–5 km	1214 (35.0)
	5–10 km	1850 (53.3)
	⩾10 km	138 (4.0)

Car and bike access	Cars per adult in household	No cars	508 (14.8)
	<1 Car per adult	1283 (37.4)
	⩾1 Cars per adult	1641 (47.8)
Any adult bike in household	No	1377 (42.2)
	Yes	1888 (57.8)

*Notes*: Numbers add to less than 3474 in some variables because of missing data. Note that the order in which the levels of household income and population density are presented has been reversed so that these variables run in the same direction as the other socio-economic and environmental variables.

a Collapsed into White/non-White in regression analyses because of small cell sizes.

**Table 2:** Individual and environmental predictors of total CO_2_ emissions from motorised travel, car CO_2_ allocated between drivers and passengers (*N* = 3474).

Variable	Level	Median	Regression coefficients (βs) and 95% CI for standardised log-transformed carbon			
Min-adjusted^a^	Multivariable 1	Multivariable 2	Multivariable 3
	*R*^2^ = 0.22	*R*^2^ = 0.29	*R*^2^ = 0.38
Sex	Female	15.7	0⁎⁎⁎	0⁎⁎⁎	0⁎	0⁎⁎⁎
Male	23.7	0.17 (0.09, 0.26)	0.15 (0.06, 0.23)	0.09 (0.01, 0.17)	0.12 (0.05, 0.19)

Age	18–34 years	14.8	0⁎⁎⁎	0⁎	0⁎⁎	0⁎⁎
35–49 years	26.6	0.38 (0.26, 0.50)	0.10 (−0.01, 0.22)	0.08 (−0.03, 0.20)	0.09 (−0.01, 0.19)
50–64 years	22.8	0.29 (0.18, 0.41)	0.17 (0.05, 0.29)	0.21 (0.08, 0.33)	0.20 (0.09, 0.31)
>65 years	13.6	−0.11 (−0.23, 0.01)	0.09 (−0.07, 0.25)	0.12 (−0.04, 0.28)	0.14 (−0.01, 0.30)

Ethnicity	White	18.8	0	0	0	0
Non-white	16.9	−0.16 (−0.42, 0.09)	0.02 (−0.23, 0.27)	0.06 (−0.18, 0.29)	0.10 (−0.12, 0.31)

Any child under 16	No	17.2	0⁎	0	0	0
Yes	24.6	0.14 (0.02, 0.26)	0.07 (−0.04, 0.19)	0.09 (−0.02, 0.19)	0.03 (−0.07, 0.13)

Education	Degree	24.9	0⁎⁎⁎	0⁎⁎⁎	0⁎⁎⁎	0⁎
A-level	18.6	−0.34 (−0.47, −0.20)	−0.11 (−0.24, 0.01)	−0.10 (−0.22, 0.02)	−0.08 (−0.20, 0.03)
GCSE	17.7	−0.35 (−0.47, −0.23)	−0.19 (−0.30, −0.07)	−0.19 (−0.30, −0.08)	−0.10 (−0.20, 0.00)
No formal	12.2	−0.56 (−0.68, −0.44)	−0.27 (−0.39, −0.16)	−0.25 (−0.36, −0.13)	−0.17 (−0.28, −0.05)

Annual household income	>£40,000	31.3	0⁎⁎⁎	0⁎⁎⁎	0⁎⁎⁎	0⁎
£20–40,000	21.7	−0.22 (−0.33, −0.11)	−0.10 (−0.21, 0.01)	−0.03 (−0.13, 0.06)	−0.02 (−0.11, 0.08)
<£20,000	10.7	−0.71 (−0.83, −0.59)	−0.31 (−0.43, −0.19)	−0.23 (−0.34, −0.12)	−0.15 (−0.25, −0.05)

Housing tenure	Owned	22.2	0⁎⁎⁎	0⁎⁎⁎	0⁎⁎⁎	0
Privately rented	9.8	−0.55 (−0.70, −0.41)	−0.35 (−0.50, −0.21)	−0.19 (−0.33, −0.05)	−0.07 (−0.20, 0.06)
Council rented	5.6	−0.91 (−1.07, −0.76)	−0.53 (−0.69, −0.37)	−0.43 (−0.59, −0.27)	−0.11 (−0.26, 0.04)
Other	14.3	−0.06 (−0.29, 0.17)	0.06 (−0.14, 0.26)	0.07 (−0.12, 0.27)	0.17 (−0.01, 0.34)

Employment status	Full-time	31.3	0⁎⁎⁎	0⁎⁎⁎	0⁎⁎⁎	0⁎⁎⁎
Part-time	20.4	−0.26 (−0.36, −0.15)	−0.21 (−0.31, −0.11)	−0.13 (−0.23, −0.03)	−0.14 (−0.23, −0.04)
Student	4.3	−0.90 (−1.12, −0.68)	−0.73 (−0.95, −0.51)	−0.68 (−0.89, −0.47)	−0.52 (−0.72, −0.32)
Retired	13.8	−0.52 (−0.65, −0.39)	−0.40 (−0.53, −0.28)	−0.15 (−0.31, 0.00)	−0.16 (−0.32, −0.01)
Home duties	13.5	−0.54 (−0.74, −0.33)	−0.41 (−0.60, −0.23)	−0.25 (−0.46, −0.05)	−0.19 (−0.38, −0.01)
Other	5.8	−1.01 (−1.19, −0.83)	−0.66 (−0.84, −0.47)	−0.44 (−0.64, −0.24)	−0.32 (−0.52, −0.13)

Site	Southampton	12.1	0⁎⁎⁎		0	0
Cardiff	19.5	0.26 (0.16, 0.36)		0.04 (−0.09, 0.16)	0.04 (−0.07, 0.16)
Kenilworth	23.8	0.43 (0.32, 0.54)		0.11 (−0.02, 0.24)	0.12 (0.00, 0.24)

Urban/rural status	Urban	18.1	0⁎⁎⁎		0⁎	0⁎
Rural	32.3	0.47 (0.32, 0.61)		0.21 (0.04, 0.39)	0.18 (0.02, 0.34)

Population density	Change per 10 people per hectare	–	−0.05 (−0.06, −0.03)⁎⁎⁎		−0.01 (−0.02, 0.01)	0.00 (−0.02, 0.01)

Home-work distance	0–2 km or did not commute	11.2	−0.18 (−0.31, −0.04)		−0.05 (−0.19, 0.09)	−0.05 (−0.18, 0.08)
2–5 km	13.8	0⁎⁎⁎		0⁎⁎⁎	0⁎⁎⁎
5–10 km	25.7	0.40 (0.24, 0.56)		0.25 (0.09, 0.41)	0.20 (0.06, 0.33)
10–20 km	36.1	0.77 (0.59, 0.94)		0.56 (0.41, 0.70)	0.46 (0.32, 0.60)
⩾20 km or variable	68.3	0.92 (0.76, 1.08)		0.77 (0.63, 0.91)	0.67 (0.54, 0.81)

Home-retail distance	Change per kilometer	−	0.08 (0.06, 0.09)⁎⁎⁎		0.00 (−0.03, 0.03)	−0.01 (−0.04, 0.01)

Cars per adult in household	No cars	3.0	−0.97 (−1.10, −0.84)			−0.75 (−0.88, −0.61)
<1 Car per adult	14.9	0⁎⁎⁎			0⁎⁎⁎
⩾1 Cars per adult	28.6	0.52 (0.44, 0.59)			0.32 (0.25, 0.39)

Any adult bike	No	15.7	0			0⁎⁎
Yes	21.7	0.01 (−0.08, 0.11)			−0.12 (−0.20, −0.04)

⁎ *p* < 0.05.

⁎⁎ *p* < 0.01.

⁎⁎⁎ *p* < 0.001.

a Minimally-adjusted analyses adjust for age and sex only, multivariable analyses adjust for all variables in column. The dependent variable is kilograms of CO_2_ per week transformed as log([CO_2_/mean(CO_2_)] + 0.01), meaning the unit of analysis is standard deviations of log-transformed CO_2_ and dimensionless.

**Table 3:** Individual and environmental predictors of transport CO_2_ emissions for different journey purposes, car CO_2_ shared between drivers and passengers (*N* = 3474).

Variable	Level	Regression coefficients (βs) and 95% CI for standardised log-transformed carbon			
Commuting	Business	Shopping/personal	Social/leisure
*R*^2^ = 0.54	*R*^2^ = 0.14	*R*^2^ = 0.18	*R*^2^ = 0.12
Sex	Female	0	0⁎⁎⁎	0	0
Male	0.02 (−0.04, 0.08)	0.18 (0.10, 0.26)	−0.04 (−0.12, 0.04)	−0.03 (−0.11, 0.04)

Age	18–34 years	0⁎	0⁎	0⁎	0
35–49 years	0.04 (−0.04, 0.13)	0.08 (−0.05, 0.22)	0.11 (−0.01, 0.23)	−0.04 (−0.16, 0.08)
50–64 years	0.02 (−0.06, 0.11)	0.20 (0.06, 0.34)	0.19 (0.06, 0.32)	−0.03 (−0.16, 0.09)
>65 years	−0.10 (−0.23, 0.02)	0.24 (0.07, 0.40)	0.20 (0.03, 0.38)	−0.04 (−0.20, 0.13)

Ethnicity	White	0⁎	0	0	0⁎⁎⁎
Non-white	0.21 (0.05, 0.38)	0.06 (−0.11, 0.22)	0.01 (−0.18, 0.19)	−0.28 (−0.44, −0.12)

Any child under 16	No	0	0	0	0
Yes	0.05 (−0.04, 0.13)	0.06 (−0.07, 0.18)	0.08 (−0.04, 0.20)	−0.02 (−0.13, 0.09)

Education	Degree	0	0	0	0⁎⁎⁎
A-level	0.01 (−0.08, 0.11)	−0.05 (−0.16, 0.07)	0.00 (−0.12, 0.11)	0.06 (−0.05, 0.18)
GCSE	0.02 (−0.06, 0.10)	0.01 (−0.11, 0.13)	−0.04 (−0.14, 0.06)	−0.15 (−0.26, −0.04)
No formal	0.05 (−0.03, 0.13)	−0.10 (−0.20, 0.01)	−0.14 (−0.25, −0.03)	−0.17 (−0.28, −0.06)

Annual household income	>£40,000	0	0⁎⁎	0	0
£20–40,000	−0.02 (−0.11, 0.06)	−0.14 (−0.24, −0.04)	0.10 (0.00, 0.20)	0.02 (−0.08, 0.13)
<£20,000	−0.07 (−0.16, 0.03)	−0.17 (−0.26, −0.07)	0.03 (−0.09, 0.14)	−0.06 (−0.17, 0.05)

Housing tenure	Owned	0	0	0	0
Privately rented	0.00 (−0.11, 0.11)	−0.04 (−0.17, 0.10)	−0.07 (−0.20, 0.06)	−0.03 (−0.16, 0.10)
Council rented	0.10 (−0.02, 0.21)	−0.08 (−0.20, 0.04)	−0.03 (−0.18, 0.13)	−0.09 (−0.23, 0.06)
Other	0.16 (0.03, 0.30)	−0.05 (−0.28, 0.17)	−0.11 (−0.38, 0.16)	0.19 (−0.06, 0.43)

Employment status	Full-time	0⁎⁎⁎	0⁎⁎⁎	0⁎⁎⁎	0⁎⁎⁎
Part−time	−0.11 (−0.21, −0.02)	−0.21 (−0.34, −0.09)	0.04 (−0.07, 0.15)	0.09 (−0.02, 0.20)
Student	−0.33 (−0.49, −0.17)	−0.47 (−0.61, −0.33)	−0.43 (−0.61, −0.24)	−0.25 (−0.43, −0.07)
Retired	−0.66 (−0.78, −0.54)	−0.73 (−0.85, −0.60)	0.36 (0.21, 0.50)	0.29 (0.13, 0.45)
Home duties	−0.51 (−0.70, −0.32)	−0.55 (−0.69, −0.42)	0.26 (0.07, 0.44)	0.12 (−0.08, 0.32)
Other	−0.57 (−0.72, −0.41)	−0.39 (−0.56, −0.21)	0.08 (−0.12, 0.28)	0.02 (−0.17, 0.22)

Site	Southampton	0	0	0	0
Cardiff	0.07 (−0.03, 0.16)	0.04 (−0.07, 0.14)	0.04 (−0.08, 0.16)	0.03 (−0.09, 0.15)
Kenilworth	0.10 (0.00, 0.20)	0.08 (−0.05, 0.20)	0.09 (−0.04, 0.22)	0.03 (−0.11, 0.17)

Urban/rural status	Urban	0	0	0	0^∗^
Rural	0.03 (−0.15, 0.22)	−0.04 (−0.25, 0.17)	0.08 (−0.14, 0.30)	0.22 (0.00, 0.43)

Population density	Change per 10 people per hectare	0.00 (−0.01, 0.01)	0.00 (−0.01, 0.02)	−0.01 (−0.02, 0.01)	−0.01 (−0.02, 0.01)

Home-work distance	0–2 km/no commute	−0.34 (−0.45, −0.24)	0.02 (−0.10, 0.14)	0.04 (−0.09, 0.17)	0.02 (−0.10, 0.14)
2–5 km	0⁎⁎⁎	0	0	0
5–10 km	0.40 (0.29, 0.50)	0.00 (−0.14, 0.14)	0.03 (−0.12, 0.18)	−0.04 (−0.17, 0.10)
10–20 km	0.67 (0.57, 0.77)	0.07 (−0.11, 0.25)	0.02 (−0.15, 0.19)	0.08 (−0.09, 0.25)
⩾20 km or variable	0.81 (0.68, 0.94)	0.11 (−0.06, 0.27)	0.10 (−0.05, 0.25)	0.01 (−0.14, 0.16)

Home-retail distance	Change per kilometers	−0.01 (−0.03, 0.01)	−0.01 (−0.03, 0.02)	0.02 (−0.01, 0.04)	−0.01 (−0.04, 0.02)

Cars per adult in household	No cars	−0.33 (−0.42, −0.24)	0.00 (−0.10, 0.10)	−0.60 (−0.72, −0.48)	−0.47 (−0.59, −0.36)
<1 Car per adult	0⁎⁎⁎	0⁎⁎⁎	0⁎⁎⁎	0⁎⁎⁎
⩾1 Cars per adult	0.20 (0.14, 0.26)	0.21 (0.12, 0.30)	0.11 (0.03, 0.20)	0.25 (0.17, 0.34)

Any adult bike	No	0	0⁎	0⁎⁎	0
Yes	−0.03 (−0.10, 0.03)	−0.09 (−0.19, 0.00)	−0.12 (−0.21, −0.03)	−0.02 (−0.10, 0.07)

^a^ Multivariable analyses adjust for all variables in column. The dependent variable is kilograms of CO_2_ per week transformed as log([CO_2_/mean(CO_2_)] + 0.01), meaning the unit of analysis is standard deviations of log-transformed CO_2_ and dimensionless.

⁎ *p* < 0.05.

⁎⁎ *p* < 0.01.

⁎⁎⁎ *p* < 0.001.

## Appendix A Calculation of CO_2_ emissions from motorised travel

As described in the main text we used a detailed seven-day recall instrument to measure past-week travel behaviour across a range of modes. For travel by bus, train, and other non-car modes (taxi, ferry, underground, motorcycle and mobility scooter), we calculated the total distance travelled in the past week and multiplied this by mode-specific, average emissions factors included in UK Government 2010 guidelines for greenhouse gas reporting [33]. We excluded the flights reported by 17 participants (0.5%) because our survey instrument was not designed to capture CO_2_ emissions from air travel and because, unlike for land travel, past-week air travel is unlikely to be a good proxy for average air travel.

For cars and vans we further calibrated the process of estimating CO_2_ emissions by using participants’ reports of their time spent travelling to calculate average travel speed as a proxy for road types (e.g. urban, rural, motorway). This allowed us to apply the speed-emissions factors underlying the National Atmospheric Emissions Inventory [48] based on vehicle type approval test data. These speed-emissions curves are polynomial functions of emissions as a function of average speed, vehicle type (car or van), fuel type and propulsion technology (petrol internal combustion engine (ICE), diesel ICE, liquid petroleum gas ICE, petrol hybrid electric vehicle), engine size (<1.4 l, 1.4–2 l, >2 l) and vehicle age. An uplift factor of +15% over the test-based gCO_2_/km factors was applied to take into account the combined ‘real-world’ effects on fuel consumption not already taken into account in the test-based factors, including the use of accessories, vehicle payload, poor maintenance, gradients, weather, more aggressive/harsher driving style, etc. [33]. The ‘most used vehicle’ reported by the participants was taken as the reference vehicle for the emissions analysis. Where one or more vehicle details were omitted, the average over the unreported variable was taken as the basis for emissions factors. For example, some respondents did not report engine size while providing details on fuel type and age. In these (few) cases we used the 2010 fleet average of petrol and diesel car emissions factors.

Multiplying total distance travelled by these speed-emissions factors gave us an estimate of the total ‘hot’ emissions for each vehicle when the engine was at operating temperature. As a final adjustment to this figure, we used the total number of reported trips by each participant to estimate the number of ‘cold’ starts, as cold starts generate excess emissions (i.e. over and above the ‘hot’ emissions) due to suboptimal fuel combustion. Excess ‘cold’ start emissions were calculated as a function of the ambient air temperature, average trip length and the share of the trip length running ‘cold’.

In the absence of detailed data on the shared use of cars, previous studies have allocated all CO_2_ emissions to the driver [49,50]. The advantage of this method is that it avoids overestimating the total CO_2_ contribution from car travel by allocating full emissions to both drivers and passengers. One disadvantage is that assigning zero emissions to passenger travel by car is arguably inconsistent with the fact that we did assign CO_2_ emissions for passenger travel by public transport. We were also concerned that the driver-only approach might introduce an arguably artefactual association with CO_2_ emissions if a particular socio-economic or demographic characteristic was not associated with levels of car use and CO_2_ emissions but was associated with whether an individual chose to do the driving themselves or asked their partner to do it.

We therefore explored using an alternative approach which aimed to divide emissions between car drivers and car passengers as follows. First we calculated emissions under a ‘worst case’ scenario, assuming car drivers were alone in their vehicle (i.e. receiving full emissions) and that car passengers were in a car with only them and the driver (i.e. receiving half-emissions). We then scaled both sets of emissions downwards by the ratio of [driver CO_2_ emissions/(driver + passenger CO_2_ emissions)], calculating this ratio separately for each mode (ratios 0.94 for commuting, 0.89 for educational travel, 0.96 for business travel, 0.86 for shopping/personal business, and 0.85 for social/leisure journeys). We did this in order to avoid overestimating the total CO_2_ contribution from car travel, in recognition of the fact that some fraction of drivers will have had passengers in the car (fraction estimated from the group level data) and some fraction of passengers will have been in cars with other passengers as well (fraction unknown but assumed to be the same).

This driver/passenger approach yielded very similar estimates of total CO_2_ emissions to the ‘driver-only’ approach (Pearson’s correlation 0.91) and very similar substantive findings. We therefore use the driver/passenger approach throughout the main text.

## Appendix B Linear regression results when using driver-only approach to carbon emissions allocation

See Table B.1.

## Appendix C Results of logistic regression analyses predicting to the binary variable of being in the ‘top 20%’ of carbon emissions

See Table C.1.

## Footnotes

2 Surface transport is still dominated by vehicles with internal combustion engines running on petrol (gasoline) and diesel fuels. These propulsion systems emit relatively small amounts of the non-CO_2_ greenhouse gases methane (CH_4_) and nitrous oxide (N_2_O), adding approximately 1% to total greenhouse gas emissions over and above CO_2_.

3 We used CO_2_ and not CO_2_ equivalent as our primary outcome measures because (a) CO_2_ emissions dominate direct CO_2_e emissions from surface passenger transport, making up approximately 99% of direct CO_2_e and (b) speed-emissions curves for cars and vans for the non-CO_2_ greenhouse gases methane (CH_4_) and nitrous oxide (N_2_O) are less robust than for CO_2_, thus potentially introducing uncertainty in outcome measures for little added benefit.

4 Although these aggregate emissions factors are based on real fleet and travel data they make a number of simplifying assumptions including the use of average load factors (passengers per vehicle) for UK public transport; vehicle fleet mixes in terms of age and fuel type (e.g. diesel and electric for rail); and a 15% uplift of emissions over the lab test data due to real world driving conditions.

5 We multiplied the weekly total by 47 (not 52), thus discounting 5 weeks of ‘time away from home’ (e.g. school holidays, public holidays). This was deemed appropriate since the measurement week fell outside those periods.

6 Note that it is also possible that car access could in some cases affect people’s ability to work and therefore their income, but we judged it likely that the direction of causality most often ran in the opposite direction.
